# Supplementary material for: Inter-Session Reliability of Functional Near-Infrared Spectroscopy at the Prefrontal Cortex While Walking in Multiple Sclerosis
Source: Brain Sci. 2020 Sep 17;10(9):643. doi: 10.3390/brainsci10090643 (PMC7565127; doi:10.3390/brainsci10090643)
Supplement: Supplementary file 1 [file brainsci-10-00643-s001.docx]

| **Channel** | **Position** | **Source** | **Detector** | **X [mm]** | **Y [mm]** | **Z [mm]** | **Landmark** | **Specificity [%]** | **Distance [mm]** |
| --- | --- | --- | --- | --- | --- | --- | --- | --- | --- |
| 1 | F4-F2 | F4 | F2 | 30 | 40 | 41 | 9 - Dorsolateral prefrontal cortex | 68,36664315 | 30 |
|  |  |  |  |  |  |  | 46 - Dorsolateral prefrontal cortex | 22,39597886 |  |
|  |  |  |  |  |  |  | 8 - Includes Frontal eye fields | 5,568764573 |  |
|  |  |  |  |  |  |  |  |  |  |
| 2 | F4-F6 | F4 | F6 | 46 | 38 | 24 | 45 - pars triangularis Broca's area | 70,67253504 | 30 |
|  |  |  |  |  |  |  | 46 - Dorsolateral prefrontal cortex | 23,4516285 |  |
|  |  |  |  |  |  |  |  |  |  |
| 3 | AF8-F6 | AF8 | F6 | 48 | 46 | 5 | 45 - pars triangularis Broca's area | 43,87699256 | 33 |
|  |  |  |  |  |  |  | 46 - Dorsolateral prefrontal cortex | 43,17605645 |  |
|  |  |  |  |  |  |  | 47 - Inferior prefrontal gyrus | 5,07367444 |  |
|  |  |  |  |  |  |  |  |  |  |
| 4 | AF8-Fp2 | AF8 | Fp2 | 34 | 59 | -2 | 10 - Frontopolar area | 31,0841122 | 30 |
|  |  |  |  |  |  |  | 11 - Orbitofrontal area | 30,472822 |  |
|  |  |  |  |  |  |  | 46 - Dorsolateral prefrontal cortex | 20,4016318 |  |
|  |  |  |  |  |  |  |  |  |  |
| 5 | AF4-F2 | AF4 | F2 | 22 | 52 | 33 | 9 - Dorsolateral prefrontal cortex | 51,5248078 | 44 |
|  |  |  |  |  |  |  | 46 - Dorsolateral prefrontal cortex | 26,41541438 |  |
|  |  |  |  |  |  |  | 10 - Frontopolar area | 18,40538603 |  |
|  |  |  |  |  |  |  |  |  |  |
| 6 | AF4-F6 | AF4 | F6 | 40 | 50 | 16 | 46 - Dorsolateral prefrontal cortex | 47,36091434 | 45 |
|  |  |  |  |  |  |  | 45 - pars triangularis Broca's area | 30,60393436 |  |
|  |  |  |  |  |  |  | 10 - Frontopolar area | 18,97467131 |  |
|  |  |  |  |  |  |  |  |  |  |
| 7 | AF4-Fp2 | AF4 | Fp2 | 25 | 63 | 9 | 10 - Frontopolar area | 68,78124608 | 28 |
|  |  |  |  |  |  |  | 11 - Orbitofrontal area | 21,56708753 |  |
|  |  |  |  |  |  |  | 46 - Dorsolateral prefrontal cortex | 6,609548215 |  |
|  |  |  |  |  |  |  |  |  |  |
| 8 | AF4-AFz | AF4 | AFz | 13 | 61 | 24 | 10 - Frontopolar area | 72,46582734 | 36 |
|  |  |  |  |  |  |  | 9 - Dorsolateral prefrontal cortex | 16,97337784 |  |
|  |  |  |  |  |  |  | 46 - Dorsolateral prefrontal cortex | 7,97503285 |  |
|  |  |  |  |  |  |  |  |  |  |
| 9 | Fpz-Fp2 | Fpz | Fp2 | 13 | 67 | 0 | 10 - Frontopolar area | 54,45817975 | 31 |
|  |  |  |  |  |  |  | 11 - Orbitofrontal area | 44,84702617 |  |
|  |  |  |  |  |  |  |  |  |  |
| 10 | Fpz-Afz | Fpz | AFz | 1 | 64 | 14 | 10 - Frontopolar area | 87,47712753 | 40 |
|  |  |  |  |  |  |  | 9 - Dorsolateral prefrontal cortex | 5,080149043 |  |
|  |  |  |  |  |  |  |  |  |  |
| 11 | Fpz-Fp1 | Fpz | Fp1 | -12 | 67 | 0 | 10 - Frontopolar area | 54,49683736 | 30 |
|  |  |  |  |  |  |  | 11 - Orbitofrontal area | 44,89777168 |  |
|  |  |  |  |  |  |  |  |  |  |
| 12 | AF3-AFz | AF3 | AFz | -12 | 62 | 23 | 10 - Frontopolar area | 75,76225222 | 36 |
|  |  |  |  |  |  |  | 9 - Dorsolateral prefrontal cortex | 14,5484687 |  |
|  |  |  |  |  |  |  | 46 - Dorsolateral prefrontal cortex | 8,053792322 |  |
|  |  |  |  |  |  |  |  |  |  |
| 13 | AF3-Fp1 | AF3 | Fp1 | -24 | 63 | 9 | 10 - Frontopolar area | 69,63399373 | 27 |
|  |  |  |  |  |  |  | 11 - Orbitofrontal area | 20,11557888 |  |
|  |  |  |  |  |  |  | 46 - Dorsolateral prefrontal cortex | 8,793079404 |  |
|  |  |  |  |  |  |  |  |  |  |
| 14 | AF3-F5 | AF3 | F5 | -39 | 50 | 17 | 46 - Dorsolateral prefrontal cortex | 49,33992409 | 44 |
|  |  |  |  |  |  |  | 45 - pars triangularis Broca's area | 32,11689876 |  |
|  |  |  |  |  |  |  | 10 - Frontopolar area | 16,03613047 |  |
|  |  |  |  |  |  |  |  |  |  |
| 15 | AF3-F1 | AF3 | F1 | -23 | 52 | 32 | 9 - Dorsolateral prefrontal cortex | 48,43964622 | 44 |
|  |  |  |  |  |  |  | 46 - Dorsolateral prefrontal cortex | 32,04248075 |  |
|  |  |  |  |  |  |  | 10 - Frontopolar area | 16,88767141 |  |
|  |  |  |  |  |  |  |  |  |  |
| 16 | AF7-Fp1 | AF7 | Fp1 | -33 | 59 | -2 | 11 - Orbitofrontal area | 32,71174196 | 30 |
|  |  |  |  |  |  |  | 46 - Dorsolateral prefrontal cortex | 25,27140585 |  |
|  |  |  |  |  |  |  | 10 - Frontopolar area | 25,12699295 |  |
|  |  |  |  |  |  |  | 47 - Inferior prefrontal gyrus | 13,10460546 |  |
|  |  |  |  |  |  |  |  |  |  |
| 17 | AF7-F5 | AF7 | F5 | -47 | 46 | 6 | 45 - pars triangularis Broca's area | 48,7862379 | 33 |
|  |  |  |  |  |  |  | 46 - Dorsolateral prefrontal cortex | 43,20372761 |  |
|  |  |  |  |  |  |  |  |  |  |
| 18 | F3-F5 | F3 | F5 | -46 | 39 | 26 | 45 - pars triangularis Broca's area | 72,56408005 | 29 |
|  |  |  |  |  |  |  | 46 - Dorsolateral prefrontal cortex | 21,83837451 |  |
|  |  |  |  |  |  |  |  |  |  |
| 19 | F3-F1 | F3 | F1 | -31 | 39 | 41 | 9 - Dorsolateral prefrontal cortex | 66,60628263 | 29 |
|  |  |  |  |  |  |  | 46 - Dorsolateral prefrontal cortex | 24,84615413 |  |
|  |  |  |  |  |  |  |  |  |  |
| 20 | Fz-F2 | Fz | F2 | 10 | 41 | 50 | 9 - Dorsolateral prefrontal cortex | 68,93306313 | 29 |
|  |  |  |  |  |  |  | 8 - Includes Frontal eye fields | 28,89129929 |  |
|  |  |  |  |  |  |  |  |  |  |
| 21 | Fz-AFz | Fz | AFz | 2 | 50 | 39 | 9 - Dorsolateral prefrontal cortex | 61,76828691 | 40 |
|  |  |  |  |  |  |  | 10 - Frontopolar area | 20,26301996 |  |
|  |  |  |  |  |  |  | 8 - Includes Frontal eye fields | 12,14994939 |  |
|  |  |  |  |  |  |  | 32 - Dorsal anterior cingulate cortex | 5,083608014 |  |
|  |  |  |  |  |  |  |  |  |  |
| 22 | Fz-F1 | Fz | F1 | -9 | 41 | 50 | 9 - Dorsolateral prefrontal cortex | 63,16113601 | 29 |
|  |  |  |  |  |  |  | 8 - Includes Frontal eye fields | 34,73195285 |  |

Please note that this output was generated using fOLD-software (Zimeo Morais, G.A.; Balardin, J.B.; Sato, J.R. fNIRS Optodes' Location Decider (fOLD): a toolbox for probe arrangement guided by brain regions-of-interest. Scientific Reports 2018, 8, 3341, doi:10.1038/s41598-018-21716-z).
